# An Account of a Distemper, by the Common People in England Vulgarly Called the Mumps

**Published:** 1790

**Authors:** Robert Hamilton

**Affiliations:** Fellow of the Royal College of Physicians, and Physician at Lynn Regis, Norfolk


X. *An Account of a Diftemper, by the common People in England vulgarly called the Mumps. By* Robert Hamilton, *M. D. Fellow of the Royal College of Phyficians, F. R. S. Edin. and Phyfician at Lynn Regis, Norfolk.—From The Tranfactions of the Royal Society of Edinburgh,* Vol. II.

THE mumps, or what I beg leave to call angina maxillaris, is an epidemic difeafe of a very fingular nature. It has appeared fometimes to be pretty general; but this has not not been the cafe for many years in this place. It feems to be analogous to, if not the fame diftemper with, that called the branks by the common people in Scotland. In the general account of epidemics, in the firft volume of the Medical Effays of Edinburgh, a diforder is mentioned which feems to have been a flight degree of that which is the fubject of the following paper. I have had much practice in this difeafe, and indeed was once reduced to the utmoft danger by it myfelf.

In the following paper I fhall not pretend to give a fyftematic treatife on the mumps: I fhall relate what was the refult of obfervation, both in regard to the hiftory and cure of this difeafe; and as I fhall faithfully detail what I actually faw, I flatter myfelf that this account will not be unworthy of the perufal of future obfervers.

*The Hiftory of the Mumps is as follows:*

A laffitude, a heavinefs, a general reftlefs uneafinefs, not eafily defcribed, are perceived feveral days before the fwelling which characterifes the difeafe begins to appear. Thefe difagreeable feelings are attended with gentle rigors, and fome degree of fever, which, being flight, is commonly difregarded: then a ftiffnefs, nefs, with obtufe pain, is felt in one or both fides of the articulation of the lower jaw, impeding its motion, and, of courfe, maftication; which fymptoms increafing, a fwelling appears upon the parts the following day, and quickly extends to the parotid glands, the neighbouring fkin, and cellular membrane. Here, in fome, it ftops without difcolouring the fkin; and by keeping the parts moderately warm, and cautioufly avoiding the cold external air, the patient is foon freed from it, without any medical affiftance. But, when this is not the cafe, the parts affected generally redden the next day, the tumour becomes more diffufed, and fometimes increafes fo fuddenly in fize, that, on the third day from its firft appearance, it occupies the falivary glands and furrounding cellular membrane on that fide; and, if both fides are affected, the parts are fo much fwelled, and the tumour defcends fo low, that the countenance is rendered of a frightful enormous magnitude, and now deglutition becomes more or lefs impeded. All this is frequently without much pain; but moft commonly there is now a great deal, and a confiderable degree of fever. When this happens, the countenance appears florid, and a dufky eryfipelatous inflammation covers the the tumour, which is deepeſt in colour where there is the greateſt hardneſs, viz. on the parotid and maxillary glands. In many ſubjects here it ends: and it ſeems probable, from the natural reſolution of the diſeaſe, which now immediately follows, that the tumour has attained its greateſt magnitude, and the diſtemper its acmé; for, about the morning of the fourth day from the firſt appearance of the ſwelling, a diſcharge begins from the emunctories behind the ears; a dew-like ſweat, frequently in large drops, iſſues from every pore of the extended ſurface of tumour; a gentle diaphoreſis covers the body, if in bed; the inflammation abates; the ſwelling gradually leſſens; and, with theſe favourable circumſtances, the fever goes off, and the diſtemper totally diſappears about the ſixth day, if nature is not interrupted in her buſineſs. But if the tumour ſubſides ſuddenly about the fourth day, and one or both teſticles begin to ſwell, ſometimes with much pain, heat, inflammation, new rigors, and a freſh exacerbation of fever, much is to be apprehended from this new morbid appearance, and much circumſpection is required in the treatment of it. For the means employed by nature to promote the reſolution of the tumefied teſtes are exactly similar to those which take place in the termination of the tumours below the ears — a spontaneous discharge issues from the skin of the parts affected, and if this is copious and continued, and accompanied with a free perspiration from the surface of the whole body in bed, the disease ends happily without farther trouble; but if it is scanty, partial, or interrupted by accidental cold or imprudent treatment, the tumours of the testicles subside suddenly, the patient becomes restless, a fresh exacerbation of fever ensues, the head is affected, delirium follows, with convulsions and other dreadful symptoms, and sometimes death closes the scene.

It may be asked, whence does this train of symptoms arise? Is it from a tumefaction of the brain taking place in the instant of the sudden diminution of the tumours of the testes, as we have before seen happen to the testicles when the salivary glands suddenly subsided?

An extraordinary circumstance took place in two cases which came under my notice. One testicle in each person was found to be wasted away after the disease had ended; some particulars of which shall be mentioned in the sequel.

The

The pathognomic signs of the mumps may be readily gathered from the foregoing history, and are the same with little variation. The characteristic tumour under one or both ears, involving the salivary glands with more or less of a concomitant fever, is the first. If the disease is mild, it soon ends by a spontaneous sweating from the surface of the tumour; if not, the tumour (or tumours, if on both sides) subsides suddenly, accompanied with a fresh exacerbation of fever, and the testicles swell; and here it also sometimes goes no farther, but terminates by a discharge from the skin covering those parts. But if the testes suddenly subside, and a fresh exacerbation of fever appear at the same time, the brain is immediately affected, attended by a train of terrible symptoms, and death sometimes ends the conflict.

The mumps, so far as my observations extended, appeared generally confined to young men, from the age of puberty upwards to thirty years. Not many between thirty and forty fell under my care. I never knew above one man of forty attacked by this disease, and he suffered severely. Very few boys were affected, and those had the distemper mildly.

I never ſaw any of the female ſex above ten years old ſubject to this illneſs; and thoſe who fell under my care were not numerous, and generally had the diſeaſe mildly. I do not remember one inſtance of the mammæ being affected. I have, however, heard of this circumſtance, but cannot ſpeak as to the authenticity of my intelligence. But from what happens in men, it is, from analogy, moſt natural to ſuppoſe that the ovaria are more likely to be affected than the mammæ, although there is undoubtedly a wonderful ſympathy between the uterus, and we ſuppoſe its appendages, and the mammæ. On this matter, however, I ſhall not pretend to decide.

The mumps made its appearance in an epidemic form at Lynn in 1758, and remained ſeveral years afterwards. It was chiefly confined to the ſpring months. In the year 1761 it prevailed very much. Two companies of the Norfolk regiment of militia were quartered here, and put under my care. It raged more among theſe ſoldiers, in proportion to their number, than amongſt the inhabitants of the town. I was very ſeldom without ſeven or eight of theſe men upon my liſt ill of the mumps. After 1761 it began to decline. It, however, however, made its appearance in ſpring and autumn, more or leſs, as an epidemic, for ſeveral years afterwards; but the number afflicted with it became gradually leſs; and ſome ſporadic caſes were to be met with many years after the epidemical appearance of it had ceaſed.

It muſt be ingenuouſly confeſſed, that, on the firſt appearance of this (to me) new diſeaſe, I was much at a loſs how to treat it. In vain I ſearched in many authors for its hiſtory and cure. That ſhort account given by Mr. Gooch, in his Caſes and Remarks in Surgery, publiſhed firſt about this time, was the only one I could find, and that was too defective to form from it any general method of cure of a diſeaſe much more formidable in its appearance here than that mild ſpecies of it which ſeemed to have fallen under his care, and gave way ſo readily to the antiphlogiſtic method of cure. Obſervation ſoon taught me that this plan was not only inſufficient, but hurtful; and that large evacuations, with a view to reduce the tumors and promote their diſcuſſion, did oftener harm than good; the changes which take place in a bad kind of this diſeaſe, from the ſalivary glands to the teſtes, and from theſe to the brain, appearing to be more frequent and dangerous, when evacuations evacuations were freely and copiouſly employed, than when they were ſparingly uſed, or not at all. Thus diſappointed by following the only method of cure I had ſeen, I determined to ſtudy the diſeaſe with attention, and endeavour to imitate nature's operations in removing it; and had the ſatisfaction to ſee all my patients recover. As the antiphlogiſtic method had not been attended with ſucceſs, I avoided bleeding, unleſs it was indicated by an uncommonly hard and full pulſe, attended with great inflammation and pain; and even then I bled but ſparingly. Indeed, as highly inflammatory ſymptoms but rarely occurred, there was ſeldom occaſion for this evacuation. The bowels were kept open by clyſters; and ſometimes a gentle eccoprotic was neceſſary for this purpoſe, but the ſtronger cathartics were never uſed. As the diſcharge behind the ears, and the ſweating from the ſurface of the tumor, ſeemed to point out nature's principal reſources in terminating this diſeaſe, theſe were carefully encouraged, by wrapping the parts in flannel; and, if theſe diſcharges happened to ſtop, or even to leſſen, with an increaſe of feveriſh ſymptoms, bliſters were applied behind the ears, ſufficiently large to deſcend from thence over the whole ſurface of the tumours, tumours, which, by opening a difcharge from the parts immediately affected, imitated, in fome degree, that evacuation from them which nature eftablifhes to relieve herfelf; and by the influence of their irritation the difeafe feemed to refume afrefh its feat in the falivary glands, when it had in part left them, and taken poffeffion of the teftes. It was curious to obferve this fact. Sometimes, after the fubfiding of the tumid falivary glands, they have become fwelled and painful again: when this occurred, the tumours of the tefticles became lefs painful, more relaxed, and leffened in fize, whilft the brain, at the fame time, remained perfectly free from diforder; and this happened more than once in the fame perfon. It was, fometimes obferved, after the affection of the brain had taken place upon the fudden diminution of the tefticles, that the latter have again become tumid and painful, and that the brain, on this appearance of the difeafe in them, has been immediately relieved. Of this curious circumftance I have feen feveral inftances; but one was remarkably ftriking in a particular friend, to whom it occurred twice: he, however, did well, but one tefticle wafted away. Reflecting, foon after this paper was firft written, (which was feveral years after my obferva-tions were begun) on the extraordinary aptitude of this difeafe to fluctuate in this manner, I conceived that it would be an object of the firft confequence to fix the diftemper, if poffible, in its firft fituation, the falivary glands, until it was perfectly ended, and prevent this dange-rous difpofition of it to fhift its abode. An early irritation on the parts, and difcharge from the furface, appeared, from what had been ob-ferved of the difeafe, and its mode of termina-tion, naturally to be the moft likely means of effecting this; and blifters, from what already had been experienced, feemed to be beft calcu-lated for this purpofe. There could be no ha-zard in the trial. With this view, blifters of a fize fufficient to cover the fkin of the tumours, fuppofing they fhould afterwards attain any con-fiderable magnitude, were applied over the fali-vary glands, before the fwellings had arrived at their height, or any fpontaneous difcharge had appeared; and fo far was the experiment at-tended with fuccefs, that I do not remember a fingle inftance of a fwelling of the tefticles ta-king place where this mode of attempting to keep up the tumefaction of the falivary glands, and anticipating the natural difcharge, was put in in execution. Wherefore it became my constant practice afterwards to apply large blisters on the tumours as soon as they were sufficiently formed to characterise the disease, and I had great satisfaction in observing their utility. From analogy, we may presume, that a similar mode of practice would be attended with the most beneficial effects in cases where the tumefactions of the testicles suddenly subside, and the brain becomes affected. I never had occasion to try this; but I am so convinced, that, in cases of this kind, (where there is generally danger) it would be of the greatest advantage, that I should not hesitate a moment in covering the whole scrotum with a blister, or, rather, a blistering cataplasm, as soon as the least symptom of the head's becoming disordered appeared, with a view to recal (if I may use the expression) the disease from the brain to the testicles, whilst, to relieve the latter, epispastics should, at the same time, be fully employed over the tumefied salivary glands.

As the patients were generally relieved by a spontaneous sweating in bed, diaphoretics of spiritus mindereri, &c., with warm drinks, assisted to keep up the discharge for a day or two, and the distemper was soon at an end. If, about the the third day, the tefticles began to fwell, without any remarkable increafe of fever, the fame method relieved them: but, if this was accompanied with a low, running, quick pulfe, and reftleffnefs or anxiety, more epifpaftics were applied, and the vis vitæ kept up by neurotic cordials, befide fudorifics, with a neceffary proportion of the beft of all cordials, wine, and a plentiful fweating was encouraged. The medicines employed were various, according to circumftances, and were compofed of camphor, volatile alcali, fp. mindereri, vin. antimonial. decoct. ferpentar. &c. with a requifite proportion of opium to abate the reftleffnefs. The tumefied tefticles were fufpended in a bag-trufs; the colon was emptied by clyfters, if the patients were coftive; and, with this treatment, the patients generally got well about the fixth, feventh, or eighth day.

It is requifite here to obferve, that, although the parts affected were kept warm, and the body covered fo as to encourage a difcharge from the fkin, it was neceffary that the lungs fhould have a frequent fupply of cool frefh air; for which purpofe the curtains of the bed were kept open, and a free ventilation occafionally admitted by the door and windows of the chambers bers of the ſick, which had very beneficial effects.

In the ſpring of the year 1758, a gentleman, about twenty-two years of age, (of this town) of a plethoric habit, was ſeized with the mumps. The tumours of the parotid and maxillary glands were large, hard, and inflamed, and accompanied with much fever. He was bled copiouſly, and took a briſk cathartic, which produced very frequent and large evacuations. The tumours ſuddenly ſubſided, and his teſticles as ſuddenly ſwelled to an enormous ſize, attended with great pain and much fever. Unfortunately, the laſt tumefaction was ſuſpected to ariſe from a venereal cauſe: the event fatally proved the contrary. In conſequence of the opinion of its being venereal, plentiful evacuations were deemed neceſſary; and accordingly bleeding and briſk purging were again repeated. The cataſtrophe was dreadful; for the ſwelled teſticles ſubſided ſuddenly the next day, the patient was ſeized with a moſt frantic delirium, the nervous ſyſtem was ſhattered with ſtrong convulſions, and he died raving mad the third day after. My affairs calling me abroad, prevented my being preſent on this occaſion; but on my return, at the end of the ſame year,

I received this account from a late learned and worthy phyſician* who attended the patient the three laſt days of his diſeaſe.

I had not many inſtances where the brain was affected. One, however, of a gentleman, in April, 1762, was marked with a circumſtance ſo extraordinary, that I beg leave to give ſome account of it. This perſon was about forty years of age, of a full habit, and had been bled ſome days before any ſymptom of the mumps appeared. He was obliged to travel a journey in a chaiſe the ſecond day after the parotid glands began to ſwell. On the day following this the tumours of the ſalivary glands had greatly increaſed, were inflamed, and the patient had much fever. On the morning of the fourth day the ſwellings were very much enlarged, and the teſticles began to be affected with pain: on the evening of that day the right one ſwelled. On the fifth day both teſticles were much tumefied; but the right one was by far the moſt ſo, and ſoon became twice the ſize of the other; and the ſalivary glands were found to be very conſiderably diminiſhed. On the day following this the teſticles were found

* Dr. Joſ. Tayler.

leſſened in ſize, and the patient was become reſtleſs, delirious, with much fever, and had paſſed a very bad night; yet the teſticles did not ſpeedily, nor altogether, ſubſide after the delirium began. Large bliſters, nervous and other medicines, with ſtrict confinement in bed, agreeably to the mode of cure before mentioned, ſoon relieved the patient; the teſticles ſwelled again, the delirium left him, the fever went off, and the diſeaſe gradually ceaſed. The moſt remarkable circumſtance attending this caſe was, that the right teſticle, which was twice the magnitude of the other, and was the firſt attacked, was found, after the tumours in both had ſubſided, and the diſeaſe was at an end, to be reduced to almoſt half its natural dimenſions, and kept gradually waſting, till, at length, a mere empty bag, conſiſting of the coats only, remained. The glandular body of the teſticle has been long gone; neither is the epididymis at this time (April, 1789) to be felt; the empty tunics are moſtly flaccid, but ſometimes they contract into a flattened body of an oblong ſhape, ſomewhat like an almond. This body is very tender, and gives pain when inadvertently preſſed, or touched with roughneſs, which pain ſtrikes in the inſtant up the ſpermatic tic chord to the loins, and is exquisite for a few seconds. This, however, seldom happens, as he is particularly careful to defend this very sensible and irritable part from injury; the spermatic chord is contracted, and feels hard to the touch, but this also is extremely sensible. From all which circumstances it may be presumed, that the vessels are much lessened in diameter, and perhaps the spermatic artery is become impervious; but the nerves have acquired more sensibility and irritability. After his recovery he found no other inconvenience from this extraordinary change than what we have named; has had two children since, one born in 1769, and the other in 1772, who have both healthy constitutions; and he now enjoys as good health as most men at his time of life.

Another case of a wasted testicle, in consequence of the mumps, came afterwards under my inspection.

A young man, of twenty-five years of age, of a healthy constitution, was, in the end of the year 1769, attacked by this distemper. Upon the tumid salivary glands subsiding suddenly, the testicles became affected. One of them was much more swelled than the other, and was found, when the swelling was reduced, to to be diminiſhed more than one half of its natural ſize, at which it remained in Auguſt, 1771.

Of the great number that fell under my care, there was but one caſe which terminated in ſuppuration. This was a young militia ſoldier, of about nineteen years of age, in the year 1761. The tumour was on the left ſide, of an enormous magnitude, reaching from about an inch above the maſtoid proceſs to the ſhoulder. It was opened by inciſion, and about two pints of matter were diſcharged. The ſeat of it was entirely in the cellular membrane, no ſuppuration having taken place in the ſalivary glands themſelves. There were large ſloughs of the morbid cellular membrane ſeparated from the vaſt cavity of this abſceſs; the looſe integuments united after this very ſoon to the parts beneath, and the man got well in a ſhort time.

About the end of the year 1762, the learned and ingenious Dr. Ruſſel's Œconomia Naturæ in Morbis Glandularum fell into my hands. There I was pleaſed to find an account of the mumps, and glad to ſee my obſervations ſomewhat corroborated by ſuch an authority. He thinks it contagious—"Angina hæc ex epide-"micis una eſt, et contagioſa, et per totas

"domos graffari folet, nifi antea fortaffe ju-
"venes eodem morbo laboraverint." The laft part of this fentence implies an opinion that people are not liable to have this difeafe more than once. I do not remember an inftance of a perfon's having it a fecond time. I have feen it go through a family of feveral children, which inclined me to think it contagious; but when I had the difeafe, not any one elfe in my family, which confifted of four children and fix adults, was attacked by it, although my cafe was a very bad one. About twelve months after my recovery, one of my daughters, about fix years old, had the difeafe, and all the reft efcaped: and what is ftill more remarkable, I do not remember an inftance, in the families where the militia foldiers ill of this diftemper were quartered, of a fingle perfon's being infected by them.

Dr. Ruffel, p. 116, relates a cafe of a patient deftroyed by the mumps, nearly fimilar to that given in this paper.

Hippocrates, fect. 1. book 1. of his Epidemics, appears to have defcribed the mumps. I fhall take the liberty to tranfcribe the paffage from Dr. Freind's tranflation ——

"Multis vero aurium tumores fubnafceban-
"tur, qui in alteram partem vergebant, plerif-

"que

"que etiam in utramque, iisque febre vacuis

"et in erectum stantibus nec decumbentibus,

"etsi nonnullis paulisper incalescerent; omni-

"bus absque noxa extincti sunt, neque cui-

"quam, velut ii, qui alias sui ortus causas ha-

"bent, suppurationem fecerunt. Horum au-

"tem ea fuit natura, ut molles et laxi essent,

"magni, diffusi aut sparsi, sine inflammatione

"et dolore. Omnibusque sensim et sine ulla

"significatione evanescerent. Fiebant ista qui-

"dem adolescentibus, juvenibus, ætate floren-

"tibus, atque horum plurimis qui in palæstra,

"et gymnasiis exercebantur; mulieribus vero

"paucis contingebant. Multis tusses aridæ et

"inanes, quibus cum tussi nihil educebatur,

"nec ita multo post voces raucescebant. Qui-

"busdam vero ex temporis intervallo inflam-

"mationes cum dolore in alterum testem erum-

"pebant, quibusdam etiam in utrosque. Alii

"quidem febribus corripiebantur, nonnulli

"vero sine febre persistebant. Atque adeo

"hæc ipsa plurimis gravia et molesta fuere.

"De reliquo autem quod ad ea attinet, quæ

"ad chirurgiam spectant, in his inculpate ha-

"bebant."

The spring of the year 1761 was very cold and wet; and those young militia soldiers, who were moſt liable to this diſeaſe, were out early and late in the low damp grounds adjoining this town, to learn their manual exerciſe, which correſponds with this paſſage — " Fiebant iſta " quidem adoleſcentibus, juvenibus, ætate flo- " rentibus, atque horum plurimis, qui in pa- " læſtra, et gymnaſiis exercebantur."

Tiſſot, in his *Avis au Peuple*, when treating of diſeaſes of the throat, mentions a diſtemper which is common in Switzerland, called by the French *Les Oreillons, ou Les Ourles*. This is a ſwelling of the ſalivary glands, particularly the parotids and maxillaries, and appears to be a mild ſpecies of the mumps. The tumour is ſometimes ſo large as to cauſe a difficulty in ſwallowing, and alſo to prevent the mouth from opening without pain. Children are more liable to it than adults; but as it is ſeldom attended with fever, no medicines are required. All that is neceſſary is, to protect the parts from the air, to apply a ſoft poultice, to live abſtemiouſly, eſpecially in reſpect to animal food and wine, to drink a weak warm beverage, and promote perſpiration. He ſays he cured himſelf in four days with balm tea, one fourth of milk, and a little bread in it. He does not mention any ſwelling of the teſticles; therefore this probably did not happen in Switzerland, as it never does in very ſlight caſes of this diſeaſe.

I beg leave to conclude this paper with the following words of Celſus:

"Sæpe vero etiam nova incidere genera "morborum in quibus nihil adhuc uſus oſten- "derit. Ut ideo neceſſarium ſit animadver- "tere, unde ea ceperint; ceu ſine quo nemo "mortalium reperire poſſit, cur hoc, quam "illo, potius utatur."

